# Body size and hosts of *Triatoma infestans* populations affect the size of bloodmeal contents and female fecundity in rural northwestern Argentina

**DOI:** 10.1371/journal.pntd.0006097

**Published:** 2017-12-06

**Authors:** Ricardo E. Gürtler, María del Pilar Fernández, María Carla Cecere, Joel E. Cohen

**Affiliations:** 1 Laboratorio de Eco-Epidemiología, Facultad de Ciencias Exactas y Naturales, Universidad de Buenos Aires, Buenos Aires, Argentina; 2 Instituto de Ecología, Genética y Evolución de Buenos Aires, Consejo Nacional de Investigaciones Científicas y Técnicas, Buenos Aires, Argentina; 3 Laboratory of Populations, Rockefeller and Columbia Universities, New York, New York, United States of America; 4 Earth Institute and Department of Statistics, Columbia University, New York, New York, United States of America; 5 Department of Statistics, University of Chicago, Chicago, Illinois, United States of America; University of Texas at El Paso, UNITED STATES

## Abstract

Human sleeping quarters (domiciles) and chicken coops are key source habitats of *Triatoma infestans*—the principal vector of the infection that causes Chagas disease—in rural communities in northern Argentina. Here we investigated the links among individual bug bloodmeal contents (BMC, mg), female fecundity, body length (L, mm), host blood sources and habitats. We tested whether L, habitat and host blood conferred relative fitness advantages using generalized linear mixed-effects models and a multimodel inference approach with model averaging. The data analyzed include 769 late-stage triatomines collected in 120 sites from six habitats in 87 houses in Figueroa, Santiago del Estero, during austral spring. L correlated positively with other body-size surrogates and was modified by habitat type, bug stage and recent feeding. Bugs from chicken coops were significantly larger than pig-corral and kitchen bugs. The best-fitting model of log BMC included habitat, a recent feeding, bug stage, log L_c_ (mean-centered log L) and all two-way interactions including log L_c_. Human- and chicken-fed bugs had significantly larger BMC than bugs fed on other hosts whereas goat-fed bugs ranked last, in consistency with average blood-feeding rates. Fecundity was maximal in chicken-fed bugs from chicken coops, submaximal in human- and pig-fed bugs, and minimal in goat-fed bugs. This study is the first to reveal the allometric effects of body-size surrogates on BMC and female fecundity in a large set of triatomine populations occupying multiple habitats, and discloses the links between body size, microsite temperatures and various fitness components that affect the risks of transmission of *Trypanosoma cruzi*.

## Author summary

The few bug species of Triatominae that have become domesticated are the most important vectors of human *Trypanosoma cruzi* infection, which causes Chagas disease. Evolutionary theory predicts that the fitness of hematophagous species closely adapted to human habitations should increase with feeding on human hosts and insect body length (L). Testing these hypotheses is important for a better understanding of triatomine population dynamics, identifying key productive habitats, modeling parasite transmission and designing innovative vector control strategies. This study is the first to describe the distributions of L and total individual bloodmeal contents (BMC) of triatomines over bug stages and habitats in rural villages where Chagas disease is endemic, and provides a direct link between individual BMC and female fecundity, host-feeding choices and L. L positively and significantly correlated with other body-size surrogates and was modified by habitat and host associations. Feeding on humans was associated with larger BMC and maximal female fecundity in domestic triatomine populations. Human- and chicken-fed bugs had significantly larger BMC than bugs fed on other hosts. Goat-fed bugs ranked last over all habitats, in agreement with other evidence. This study demonstrates that identifiable habitat-host associations provide nearly optimal resources and conditions for *T*. *infestans*.

## Introduction

Triatomine bugs are obligate hematophagous insects with opportunistic feeding habits on mammals and birds [1]. Fewer than 20 species of Triatominae are involved in the transmission of human *Trypanosoma cruzi* infection, the causal agent of Chagas disease [2]. Only 5–8 triatomine species have become domesticated, contact humans frequently, and are the most important from a public health standpoint [3,4]. *Triatoma infestans* and *Rhodnius prolixus* lead as the main domestic vectors of *T*. *cruzi*.

Domesticity has selective advantages related to stable habitats (human dwellings) with a stable host supply represented by humans and their companion, domestic and synanthropic animals [3]. Domestication demands adaptation to colonizing human dwellings of diverse types and blood-feeding on humans and the associated mammals or birds. This process includes encountering, biting, engorging on human blood and surviving the encounter, followed by survival, development and reproduction. Host-vector interactions are affected by the host’s defensive reactions and capacity to develop a neutralizing immune response against bug saliva [5,6]. Evolutionary theory predicts that the fitness of hematophagous species that have adapted closely to human habitations should increase with adaptation to feeding on human hosts. The prediction, for the domestic mosquito *Aedes aegypti*, that “Natural selection should favor females that choose the most nutritious hosts, the trade-off here perhaps being that the most nutritious host might also be the most defensive” [7] is equally valid for triatomines.

Does blood-feeding on humans provide any fitness advantage to domestic triatomines relative to feeding on other frequent mammalian or avian host species? Recent reviews on the behavior and blood-feeding patterns of Triatominae do not provide a definite answer to whether there are host blood effects [1,8], as in mosquitoes, tsetse and sand flies [5,9]. Early studies on mass rearing of triatomines reported that the type of host blood affected the insects’ vital rates and engorgement levels [10–12]. Cohorts of *R*. *prolixus* fed artificially on citrated human and rabbit blood through a membrane during their entire lives achieved faster development and much larger bloodmeal size, total body weight (W) and female fecundity than bugs fed similarly on chicken, sheep and horse blood [13]. The nutritional quality of avian blood may be substantially inferior to that of mammals because birds usually have much lower hemoglobin and plasma protein than clinically healthy mammals [5,9], but there are tradeoffs. Triatomines are expected to engorge more easily on chickens, which have half the hematocrit of mammals and lack platelets. Chickens' lower blood viscosity would allow increased ingestion rates, implying less time to secure a full blood meal unless feeding is interrupted, and hence reduce the risks of host-induced death [5]. Whether feeding on mammals (including humans) rather than on birds confers feeding and fitness advantages does not have a definitive answer, as a brief review of the evidence [10–17] shows (S1 Text).

Bloodmeal size (i.e., the total amount of blood ingested in a single, uninterrupted feeding bout), total individual bloodmeal contents (BMC, the total amount of blood stored in a bug’s foregut or crop regardless of whether it originated from one or several blood meals) and body size [18] are expected to affect the vital rates and fitness of insect vectors and parasite transmission [5]. For Triatominae, available estimates of bloodmeal size come from laboratory experiments [e.g., 19–21] with one exception obtained in experimental chicken houses held under natural conditions [22]. Similarly, the joint stage distributions of L, W and total body weight-to-length ratios (W:L) of *T*. *infestans* populations have rarely been investigated [23–25], and their relationship with BMC and feeding on humans remain unknown. More generally, a recent review on insect body size concluded that the frequency distributions of body L and mass of the individuals of particular species have been poorly documented [26].

*Triatoma infestans* from human sleeping quarters (domestic habitats or domiciles) mainly blood-feeds on humans, dogs, chickens and cats every 3–4 days over the austral spring-summer period in rural villages of the Argentine Chaco [27–30]. This vector species occupies a wide variety of (peri)domestic habitats in this region [31–33]. We use the term "(peri)domestic" to refer to habitats that are either domestic (e.g., domiciles) or peridomestic (e.g., goat corrals), and “site” to any exemplar of a given habitat. Peridomestic infestation was positively associated with fowl numbers in Amamá, Santiago del Estero, where the domestic densities of *T*. *infestans* increased significantly with the proportion of domestic bugs fed on chickens [34]. Chicken coops and human sleeping quarters were key source habitats when compared with other frequently infested peridomestic structures in Figueroa, Santiago del Estero: blood-feeding rates, engorgement status and female fecundity were maximal in chicken coops, submaximal in human sleeping quarters, and minimal in goat corrals [23]. These analyses, stratified by habitat and clustered by bug collection site, did not investigate the links between selected fitness components (BMC and female fecundity), individual host-feeding choices and body length L. Throughout we use L as a linear measure of body size.

Here we describe the distributions of BMC and L over triatomine stages and habitats in 120 sites from six habitats in Figueroa villages using an extended allometric equation in log-transformed form (log BMC (mg) = b*log L (mm) + a). We then tested whether L and human feeding conferred relative advantages in BMC and female fecundity (as predicted by the background theory described above) using generalized linear mixed-effects models and a multimodel inference approach with model averaging. Testing these hypotheses is important for a better understanding of triatomine population dynamics (i.e., patch occupancy and abundance), identifying key productive habitats, modeling parasite transmission, and designing innovative vector control strategies such as the use of insecticides on target domestic hosts.

## Materials and methods

### Study area

Field work was carried out in 11 neighboring rural communities with 270 houses in Figueroa Department (27° 23'S, 63° 29'W), Santiago del Estero Province, Argentina, as described elsewhere [28,32]. Most houses were made of adobe walls and thatched roofs, and had multiple peridomestic structures (kitchens, storerooms, chicken coops, goat corrals, pig corrals and granaries). ‘‘Domestic bug” refers to bugs captured in the set of contiguous human sleeping quarters and rooms that share a continuous roof structure (i.e., domestic areas or domiciles).

### Study design

A cross-sectional survey of house infestation aiming at full house coverage was conducted in October-November 2003 before an insecticide trial [32]. The research team, accompanied by Chagas disease vector control personnel, visited all houses in the study area to explain to householders the goals of the trial in clear, simple language and to request access to their premises, following protocol approved by IRB No. 00001678 (NIH registered). Residents provided oral informed consent to have their residences surveyed for triatomines and sprayed with insecticides. Skilled bug collectors searched for triatomines in all (peri)domestic sites using timed manual collections with a dislodging spray (0.2% tetramethrin, Espacial 0.2, Argentina). The site where each bug was collected was classified into 16 habitat types (formerly denominated ecotopes, now called habitats) based on its function and main local host [23]. All (peri)domestic sites positive for *T*. *infestans* among house compounds inspected from 20 to 27 October 2003 were considered eligible for detailed blood-feeding studies with emphasis on domestic sites, and bugs were processed as described [28]. Late-stage bugs (i.e., fourth and fifth instars, adult females and males) were weighed individually in an electronic balance (precision, 0.1 mg, Ohaus, Pine Brooks, NJ) and measured from clypeus to abdominal tip with a hand-held vernier caliper accurate to 0.02 mm. Late stages were individually examined for the presence of colorless urine within 8 h of capture to assess whether the insect had fed the night before (i.e. recent feeding) and to estimate daily blood-feeding rates [28]. All bugs were immediately frozen and kept at -20°C upon arrival to the laboratory in Buenos Aires.

The bloodmeal contents (if any) of each late-stage bug were prepared for testing by cutting its thorax transversally at the level of the third pair of legs; the foregut (crop) with the blood meal was extracted into a previously labeled, weighed vial and then re-weighed in the electronic balance to quantify total BMC. The chorionated eggs in the oviducts of each female were counted as female fecundity, including zero for females with no chorionated eggs.

Bloodmeal contents were tested with a direct ELISA assay against human, dog, cat, chicken, pig, goat and murid rodent (rat or mouse) antisera with high sensitivity and specificity values as described [28,35]. Reactive bugs had a positive ELISA-test result against any of the tested antisera. Unmixed blood meals were those in which only one host species was detected.

### Data management and analysis

The data analyzed in this manuscript (S1 Table) include 769 late-stage insects collected from 120 georeferenced sites in 87 house compounds examined for various attributes (S1 Fig): 544 bugs for a recent blood meal; 551 for W (in mg); 550 for L; 550 for W:L; 729 for host-feeding sources (including 34 with unidentified feedings), 726 for total bloodmeal contents, and 216 for female fecundity. All proportions reported here have attached standard errors clustered by household. In models detailed below, the total number of study cases and clusters varies owing to differing numbers of missing data for infestation, feeding, fecundity and other variables shown in S1 Table. Throughout log = log_e_. We managed data and produced graphs with Stata 14.2 [36].

We used a multimodel inference approach and information-theoretic criteria to identify the most parsimonious models that best described the data [37]. These analyses were implemented in R 3.4.0 using maximum likelihood procedures and packages MuMin [38] and lme4 [39]. For model selection, we used Akaike’s Information Criterion corrected for small samples (AIC_c_) [37]. The relative importance (RI) of each variable is defined as the sum of Akaike weights in each model in which the variable is present. Δ_*i*_ quantified the difference between model *i* and the one with the lowest AIC_c_, and Akaike weights (evidence ratio) measured the relative support of all models considered in the set. We considered only models with an evidence ratio >0.

We used random-intercept multiple regression analysis clustered by bug collection site [40] to model variation in response variables (log BMC and log L) as a function of selected predictors (see below). The explanatory variables were tested for multicollinearity using the command *collin*; after mean-centering log L by bug stage (i.e., log L_c_), variable inflation factors were < 1.8 and the condition number 15.3, indicating that the multicollinearity detected between bug stage and log L_c_ was no longer a problem. Moreover, centering continuous variables reduces the amount of multicollinearity introduced when considering interaction between variables [40].

We tested whether log L varied among habitats (a categorical variable with five levels: kitchens (the reference level), domiciles, storerooms, chicken coops, and pig corrals; goat corrals and granaries were excluded because the former lacked data on L and the latter had sparse information), bug stage (a categorical variable with four levels: fourth- and fifth-instar nymphs, males and females, where fourth instars are the reference level), a recent feeding (a binary variable indicating whether the bug blood-fed on the night before, with value 1, or not, with value 0, the reference level) and all two-way interactions among them (model 1). The sample size included 543 bugs in 64 clusters with complete information for each variable.

The allometric equation in log-transformed form (log BMC = log(a) + b*log L_c_) was used as a base model. We added to this base model the expected effects of bug stage, a recent feeding (both of which were predicted to increase BMC), habitat, and selected two-way interactions with potential relevance or evidence in favor (i.e., between habitat and recent feeding or stage or log L_c_, and between log L_c_ and stage or recent feeding) (model 2). The total sample size was 512 bugs (from 62 clusters) with complete information for each variable.

In a closely related model of log-BMC variations (model 3), we replaced habitat by host blood source (both variables were closely related for most host species) and included two-way interactions between bug stage and log L_c_ or recent feeding or host blood source, and between log L_c_ and recent feeding or host blood source. In total, 482 ELISA-reactive bugs (from 60 clusters) had unmixed blood meals and complete information for each variable.

Model 4, structurally similar to model 3 though restricted to domestic bug populations, tested for effects on log BMC of having an unmixed human blood meal (as opposed to having an unmixed blood meal on chicken or on other host), recent feeding, bug stage, log L_c_, and two-way interactions between log L_c_ and recent feeding, stage or human blood meal, and between stage and human blood meal. Domestic bugs with mixed blood meals (of which there were 10), nonreactive (16) or not tested (21) by ELISA or with no BMC recorded (25) were excluded from this analysis. Missing values for recent feeding (1.3% of the values of the variable) were removed by list-wise deletion; thus, the total sample size was 274 bugs from 45 domiciles. Finally, we tested whether the successive addition of the number of resident humans, the presence of indoor-nesting chickens (a binary variable) and bug density per site (including squared bug density) improved the fit of the model.

We used negative binomial regression with robust standard errors (command *nbreg* in Stata) to test for effects of bloodmeal source on female fecundity, which was highly overdispersed. The predictor variables were log L_c_, a recent feeding, unmixed host blood meal, and the interaction between a recent feeding and unmixed host blood meal (model 5). The sample was 95 female bugs (from 40 clusters) with complete information for each variable. In a model with a similar structure (model 6), we restricted the sample to domestic females and replaced unmixed host blood meal with an unmixed human blood meal relative to chicken or other unmixed blood source (64 females from 28 clusters).

## Results

### Body size correlates

**Fig 1:**
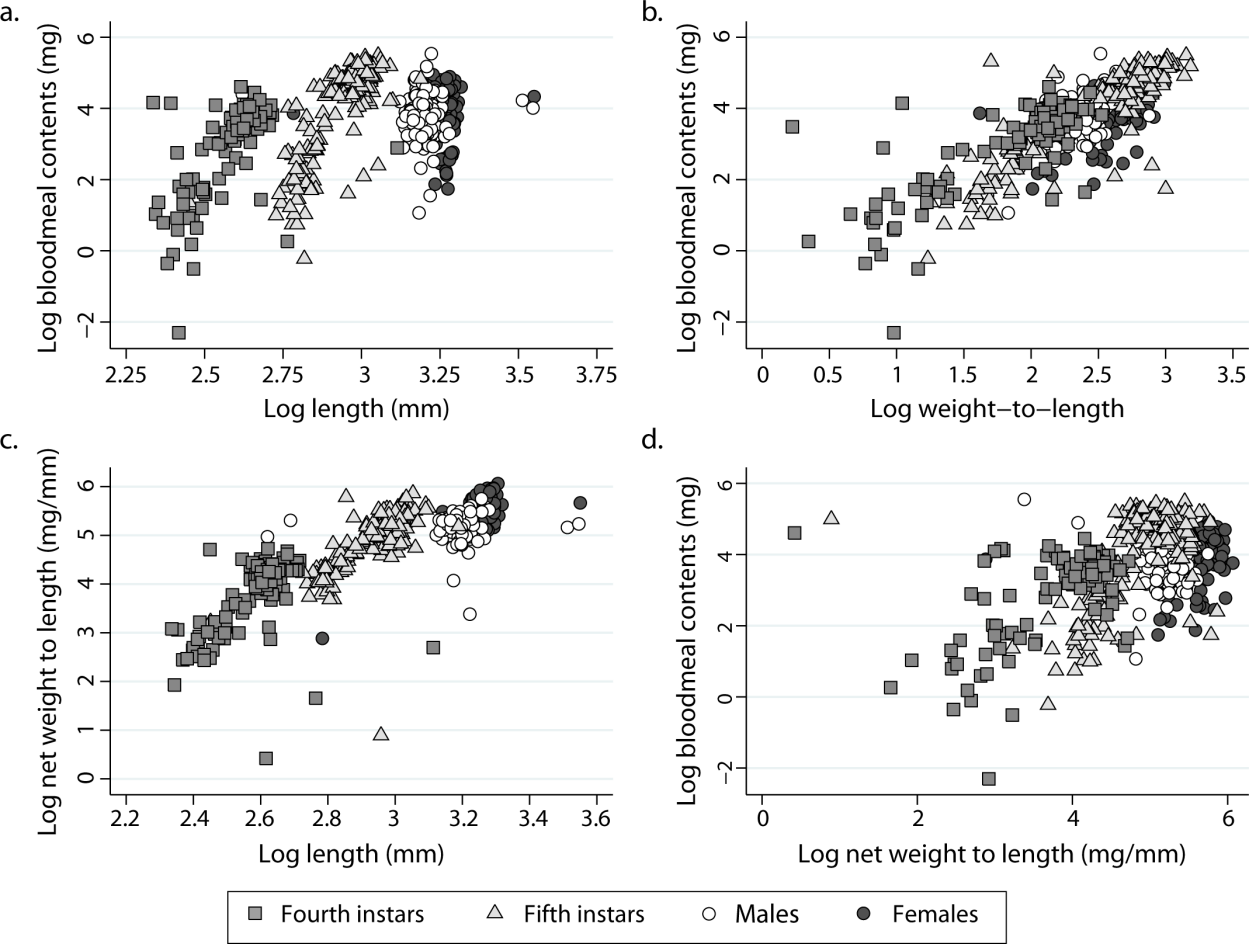
Log bloodmeal contents as a function of body length L (a) and log total weight-to-length ratios (b), and relationship between log net body weight (total body weight minus BMC) and L (c) or BMC (d) by stage of *T*. *infestans*. Figueroa, October 2003 (austral spring).

Log BMC increased linearly and highly significantly (P < 0.001) with increasing log L_c_ among fourth (r = 0.574) and fifth instars (r = 0.779), not among adult insects (Fig 1A). Table in S2 Table gives parameter estimates and other statistics. Log BMC was also highly significantly and linearly related to log W:L over all bug stages, with correlation coefficients increasing from 0.600 (males and females) to 0.777 (fourth) and 0.879 among fifth instars (Fig 1B). To avoid spurious effects from including the same quantity in the response (BMC) and predictor variables (W:L ratio), we calculated individual net body weights (i.e., W minus BMC). Log BMC correlated positively and highly significantly (P < 0.001) with log net W:L in fourth (r = 0.621) and fifth instars (r = 0.652), was weakly significant among females (r = 0.225, P = 0.020), and non-significant in male adults (r = -0.096, P > 0.25) (Fig 1D). Log net W:L correlated positively and highly significantly (P < 0.001) with log L in fourth (r = 0.590) and fifth instars (r = 0.798) and in adult females (r = 0.626), not among males (r = 0.128, P > 0.15) (Fig 1C).

The stage-specific frequency distribution of all these log-transformed variables differed highly significantly from a normal distribution (Shapiro-Wilk tests, P < 0.001). The frequency distributions of log BMC, log weight and log net weight were bimodal among fourth and fifth instars but not among adult bugs whereas log L values were right-skewed, with rare adult specimens being extremely small or extremely large (S2 Fig). We provide this information because of the scarcity of published descriptions of the distribution of insect body size.

### Body length

**Fig 2:**
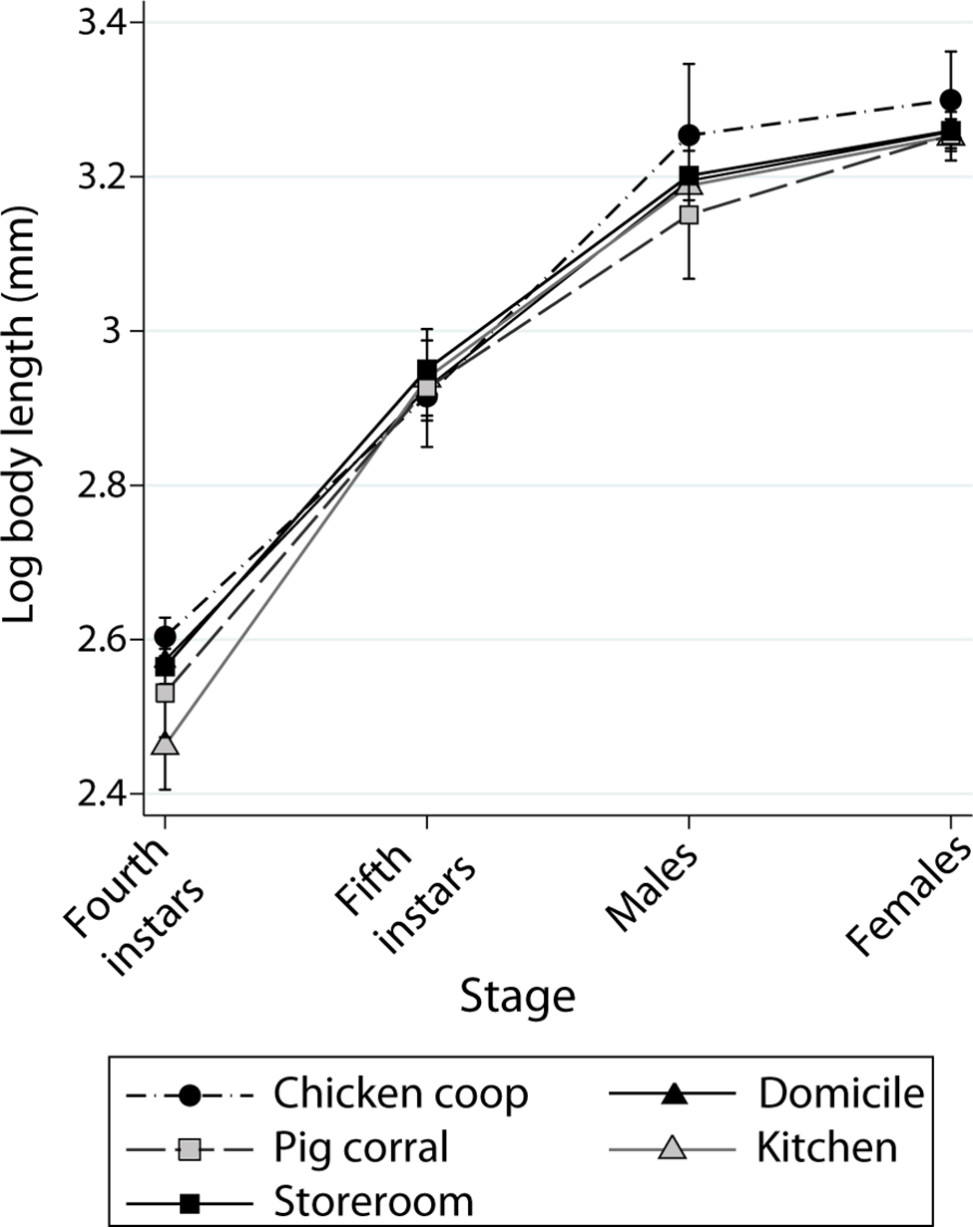
Mean body length (vertical bars are standard errors) according to bug stage and habitat in *T*. *infestans* collected in (peri)domestic habitats. Figueroa, October 2003 (austral spring).

The coefficient of variation of log L decreased with increasing bug stage from 4.4% (fourth instars), 3.4% (fifth instars), 2.6% (adult males) to 1.8% (adult females). The trajectory of mean log L across stages and habitats showed that bug populations from chicken coops nearly consistently exceeded other habitats (except for fifth instars), whereas log L scored last in bugs from pig corrals and kitchens (Fig 2). Variation in mean log L among habitats was minimal for fifth instars and females (except in chicken coops), and maximal in males. The trajectory across stages showed a consistent, positive effect of recent feeding on mean log L in the majority of habitats and nymphal stages (except in one instar from chicken coops and pig corrals), whereas effects on adults were more variable or nil and displayed some cross-overs (S3 Fig).

The best-fitting model of log L included effects of recent feeding, bug stage and their interaction (Table 1 and Table in S3 Table, model 1). The second best model also included habitat and was virtually equivalent to the best one (Δ_*i*_ = 1.75), although habitat did not exert a significant effect on log L in the averaged model (Table in S3 Table). Log L increased highly significantly with increasing bug stage and a recent feeding, as expected, and the magnitude of the effect of recent feeding on log L was higher in nymphs than in adults.

**Table 1:** Model candidates, log-likelihoods, corrected Akaike’s Information Criteria (AIC_c_), differences between model AIC_c_ and minimum AIC_c_ (Δ_*i*_), and evidence ratios (*w*_*i*_) of log body length (mm) (model 1) or log bloodmeal contents (mg) (log BMC, models 2–4) as a function of bug habitat, a recent feeding, bug stage, mean-centered log length (L_c_), host blood source, and human blood meal in *T*. *infestans* collected in (peri)domestic habitats. Figueroa, October 2003 (austral spring). Only models with an evidence ratio >0 are shown.

Model set	Variables in model	n	df	Log-likelihood	AIC_c_	Δ_*i*_	Evidence ratio (*w*_*i*_)
1	2 3 2*3	543	10	550.5	-1080.6	0	0.679
1	1 2 3 2*3	543	14	553.8	-1078.9	1.75	0.283
1	1 2 3 1*2 2*3	543	18	556.0	-1074.8	5.86	0.036
1	2 3	543	7	541.0	-1067.7	12.95	0.001
2	1 2 3 4 4*1 4*2 4*3	512	20	-562.1	1165.9	0	0.902
2	2 3 4 4*2 4*3	512	12	-573.6	1171.8	5.88	0.048
2	1 2 3 4 1*2 4*1 4*2 4*3	512	24	-560.8	1172.1	6.19	0.041
2	1 2 3 4 1*3 4*1 4*2 4*3	512	32	-554.0	1176.4	10.50	0.005
2	1 2 3 4 4*1 4*2 4*3	512	16	-571.8	1176.8	10.87	0.004
3	2 3 4 5 4*2 4*3 5*4	483	23	-455.8	960.0	0	0.608
3	2 3 4 5 2*3 4*2 4*3 5*4	483	26	-452.9	961.0	0.94	0.379
3	2 3 4 5 4*2 4*3 5*3 5*4	483	35	-446.2	968.0	8.02	0.011
3	2 3 4 5 2*3 4*2 4*3 5*3 5*4	483	38	-444.5	971.8	11.75	0.002
4	2 3 4 6 4*2 4*3 6*4	274	16	-267.6	569.3	0	0.640
4	2 3 4 6 4*2 4*3 6*3 6*4	274	22	-261.4	570.9	1.57	0.292
4	2 3 4 6 4*3 6*4	274	15	-271.3	574.4	5.06	0.051
4	2 3 4 6 4*3 6*3 6*4	274	21	-265.5	576.6	7.27	0.017

Variables: 1 Habitat; 2 Recent feeding; 3 Stage; 4 Log mean-centered body length log L_c_; 5 Host blood source; 6 Human blood meal.

### Bloodmeal contents

**Fig 3:**
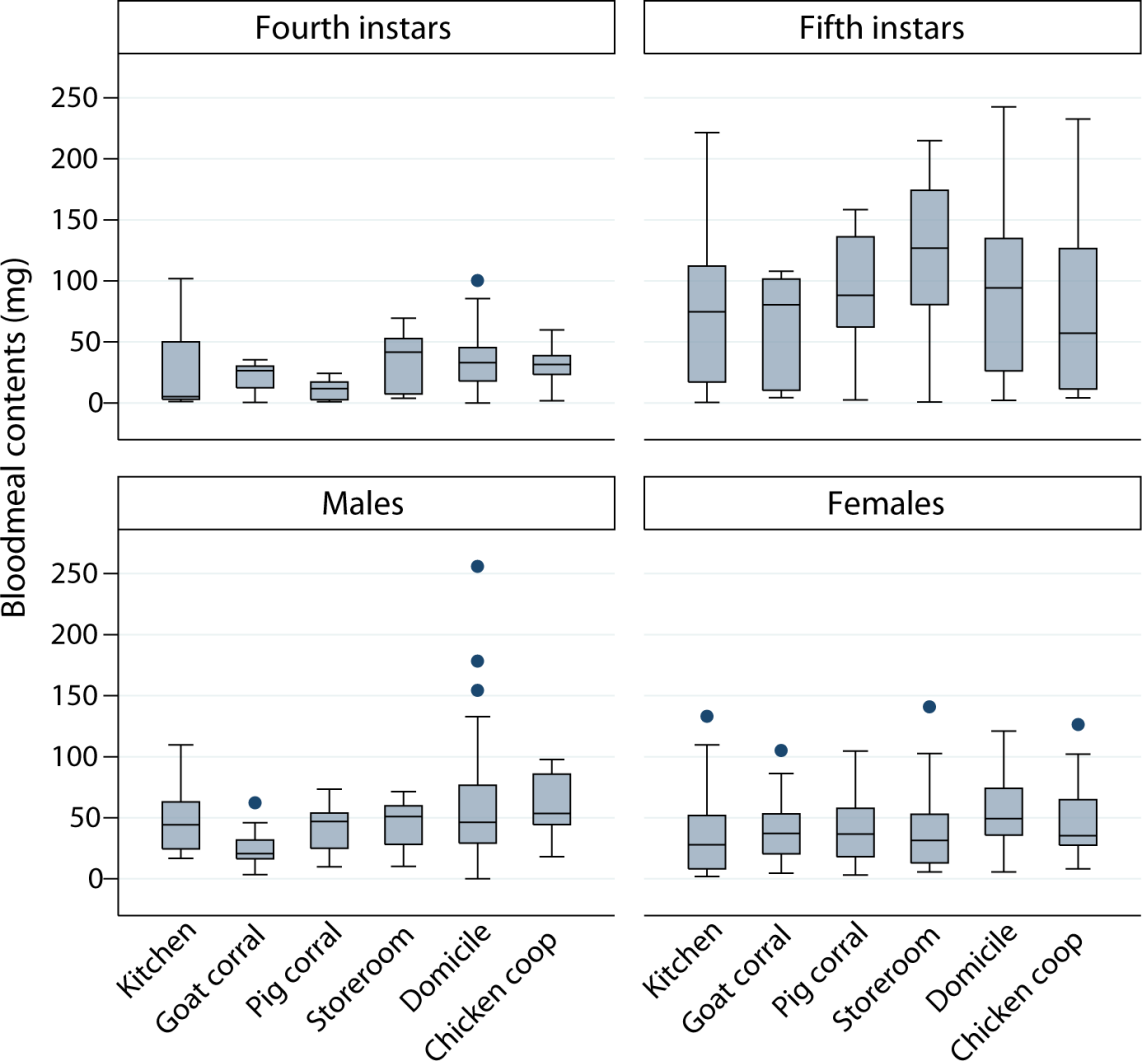
Size of bloodmeal contents of *T*. *infestans* by bug stage and habitat. Figueroa, October 2003 (spring). The boxes range from the lower quartile to the upper quartile (i.e., interquartile range). The whiskers include all data points within 1.5 of the interquartile range, and the dots are individual data points outside the range of the whiskers (outside values).

**Fig 4:**
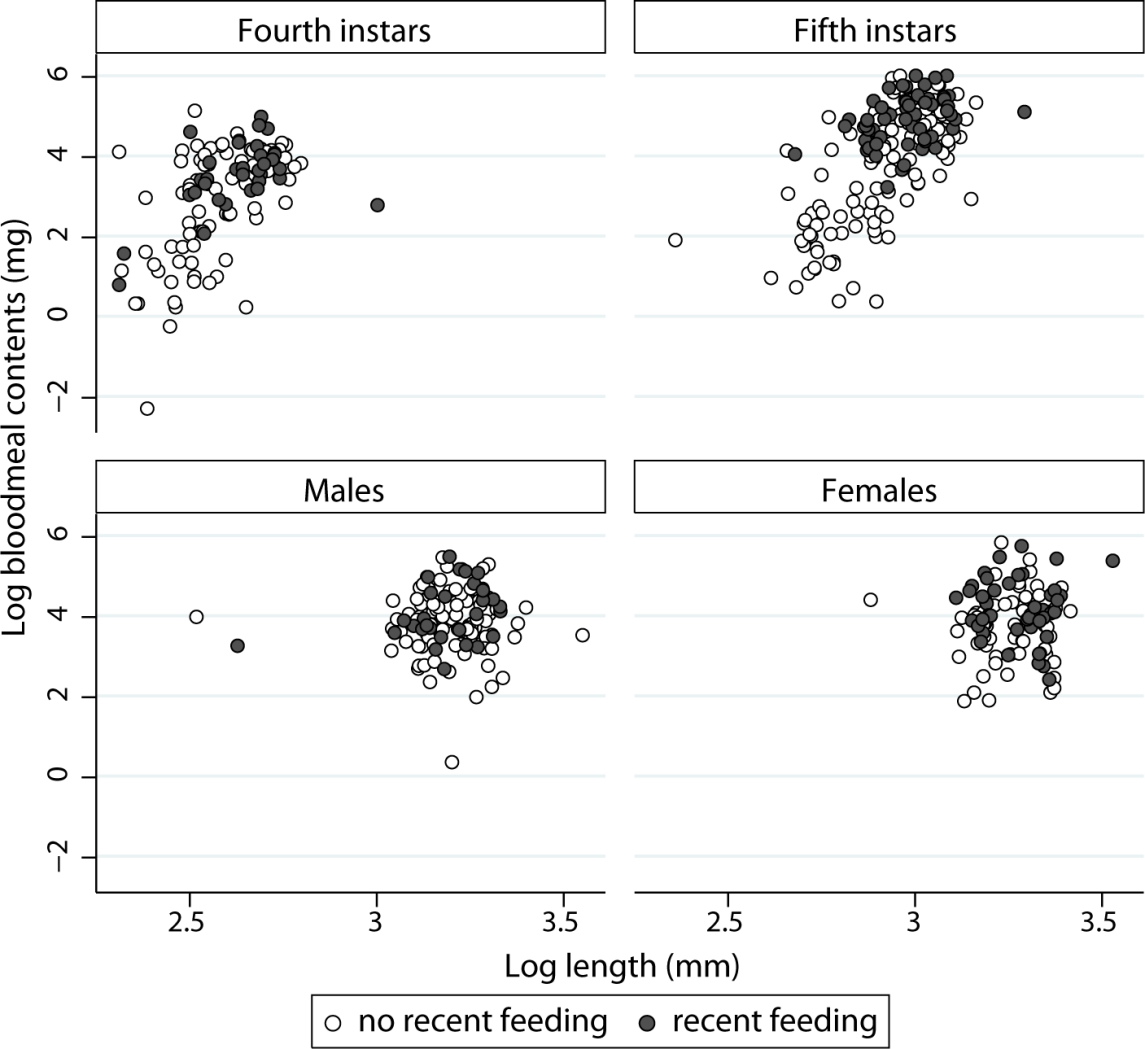
Relationship between log bloodmeal contents and log body length, a recent feeding (filled circles), and bug stage of *T*. *infestans* collected in (peri)domestic habitats; Figueroa, October 2003 (austral spring).

BMC were much larger and more variable among fifth instars than among other stages whereas only slight differences were evident among habitats except in storerooms or domiciles (Fig 3). Adult female bugs from chicken coops and domiciles showed higher median BMC than those from other habitats. In the first model set of log BMC (model 2), the best-fitting model included effects of habitat, a recent feeding, bug stage, log L_c_, and all two-way interactions between log L_c_ and other predictors (Table 1 and Table in S4 Table). Slope coefficients were highly significant for most terms except habitat, in which domiciles increased log BMC marginally significantly. There was a significant interaction between habitat and log L_c_: the effect of log L_c_ on log BMC was significantly higher in pig corrals (S4 Table). All intraclass correlation coefficients in models 2–4 were very low (ρ < 0.1), indicating very little residual heterogeneity between study sites within a habitat. For both nymphal instars, longer bugs with a recent feeding had larger BMC than shorter bugs, which were less likely to have a recent feeding (Fig 4).

**Fig 5:**
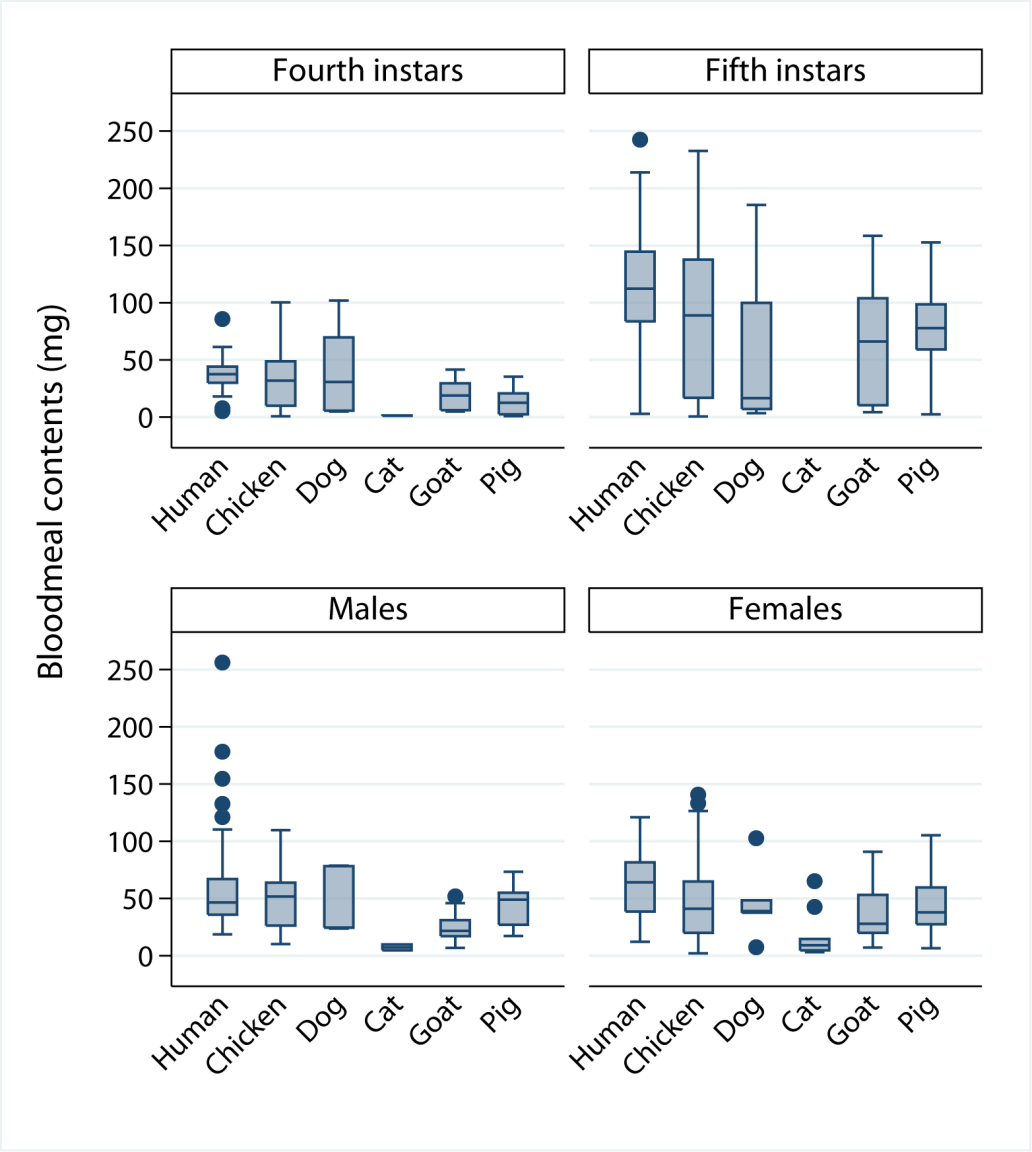
Size of bloodmeal contents of *T*. *infestans* by bug stage and unmixed host blood source. Figueroa, October 2003 (austral spring). Boxplots are as in Fig 3.

Fig 5 shows the stage-specific distribution of BMC for bugs fed on a single species of host according to host source. For all stages pooled, in domestic habitats mean BMC (in mg) peaked among human-fed bugs (70.71) and then successively decreased among chicken- (54.96), dog- (42.70) and cat-fed (13.40) bugs (Tables 2 and 3). In peridomestic habitats, mean BMC was led by chicken-fed bugs (range, 53.62–73.46) followed by pig-fed (45.77–49.33) and goat-fed bugs (31.34–51.22). Bugs fed on humans only had much larger mean BMC than bugs fed on any other host except chickens from storerooms. In the second model set of log BMC including unmixed host blood source instead of habitat (model 3), the best-fitting model included host blood source, recent feeding, bug stage, L_c_, and two-way interactions between L_c_ and all other terms included in the model (Table 1 and Table in S4 Table). The second model in this set also included the interaction between recent feeding and stage and was virtually equivalent to the best one (Δ_*i*_ = 0.94). Considering the averaged model, slope coefficients were highly significantly non-zero for most predictors except for host blood source, where its effects depended strongly on L_c_ (Table in S4 Table).

**Table 2:** Size (mg) of bloodmeal contents of *T*. *infestans* collected in human sleeping quarters according to bug stage and unmixed host-feeding source. Goat and mixed blood meals are excluded. Figueroa, October 2003 (austral spring).

	Bloodmeal contents (mg) by host blood type											
	Human			Chicken			Dog			Cat		
Stage	Mean	SE	N	Mean	SE	N	Mean	SE	N	Mean	SE	N
Fourth	37.02	2.83	33	34.26	7.18	14	17.95	9.54	4	1.3	0.2	2
Fifth	110.98	7.86	53	69.76	13.81	20	51.29	19.05	10			0
Male	60.50	5.98	53	55.21	7.64	13	45.96	13.29	5	7.3	2.6	2
Female	60.91	3.87	49	54.20	8.49	12	41.63	3.41	3	17.4	7.3	9
Total	70.71	3.56	188	54.96	5.69	59	42.70	9.38	22	13.4	5.3	13

**Table 3:** Size (mg) of bloodmeal contents of *T*. *infestans* collected in peridomestic habitats according to bug stage and unmixed host-feeding source. Human, cat^a^ and mixed blood meals are excluded. Blank cells mean, for example, that there were no goat- or pig-fed bugs in chicken coops, kitchens, or storerooms. Figueroa, October 2003 (austral spring).

		Chicken			Goat			Pig		
Habitat	Stage	Mean	SE	N	Mean	SE	N	Mean	SE	N
Chicken coop	Fourth	31.41	2.98	19						
	Fifth	75.49	11.55	33						
	Male	59.26	7.85	10						
	Female	51.43	6.44	23						
	Total	57.22	5.22	85						
Kitchen	Fourth	17.03	8.76	9						
	Fifth	79.66	10.39	28						
	Male	48.15	5.71	21						
	Female	41.23	7.71	23						
	Total	53.62	5.06	81						
Storeroom	Fourth	39.20	5.57	16						
	Fifth	137.78	13.47	23						
	Male	45.67	6.41	11						
	Female	42.20	7.28	20						
	Total	73.46	7.42	70						
Goat corral	Fourth				20.20	3.49	7	26.70	7.15	3
	Fifth				42.37	13.58	11	94.45	4.15	2
	Male				21.65	2.82	13	20.20		1
	Female				35.94	5.09	18	52.23	13.44	6
	Total				31.34	3.79	49	50.22	9.64	12
Pig corral	Fourth				12.78	4.64	4	9.90	2.99	7
	Fifth				158.40		1	87.63	15.54	9
	Male						0	45.10	4.57	14
	Female				55.45	35.45	2	48.88	5.84	21
	Total				45.77	21.82	7	49.33	4.86	51

^a^ Mean BMC (mg) for 13 bugs fed only on cats were 65.1 (storerooms), 23.7 (goat corrals), 8.3 (pig corrals), 7.1 (domiciles), 1.9 (granary) and 13.4 ± 5.3 (overall).

**Fig 6:**
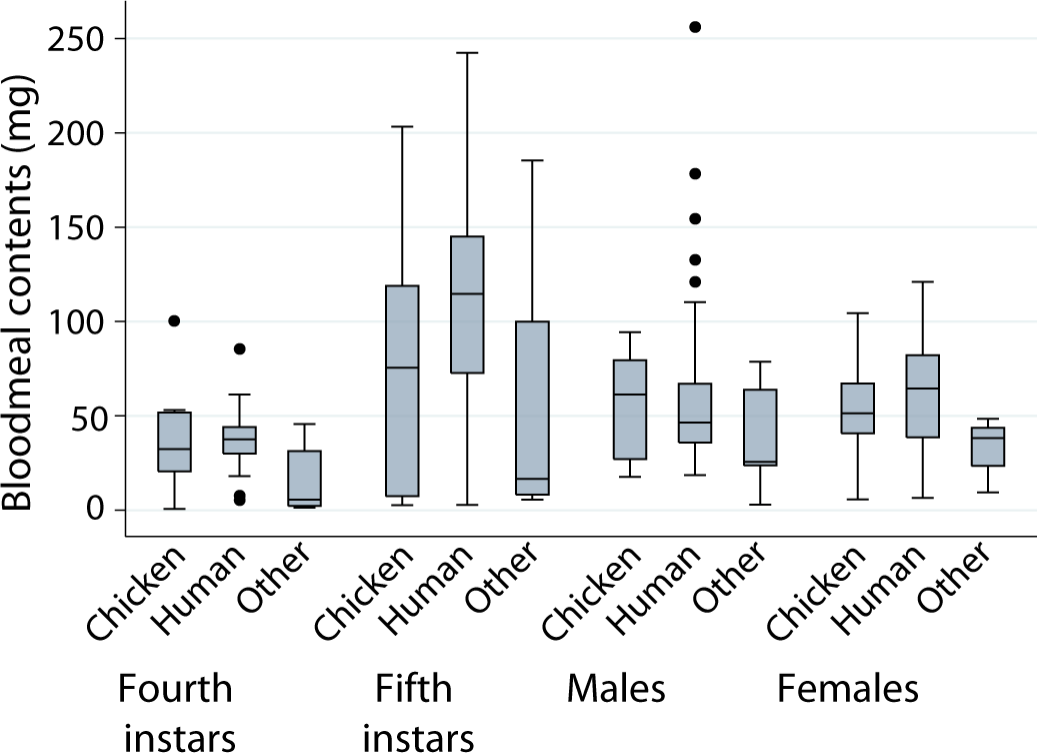
Size of bloodmeal contents of *T*. *infestans* collected in human sleeping quarters according to stage and whether they fed on only humans, only chickens or only another host (dog, cat, pig, goat or rodent). Figueroa, October 2003 (austral spring). Boxplots are as in Fig 3.

In human sleeping quarters, triatomines with an unmixed human blood meal had larger BMC than those with an unmixed blood meal on chicken or other host (Fig 6), except for male bugs. In the third model set of log BMC (model 4), the model with most support included effects of human blood meal, recent feeding, bug stage, log L_c_, and the interaction between log L_c_ and all other terms included in the model (i.e., similar to model set 2 and 3, Table 1 and Table in S4 Table). For example, the predicted BMC for human-fed only fifth instars with a recent feeding and average L was 10.43 mg greater than chicken-fed only fifth instars of similar status. The second model in this set (within 1.57 Akaike units from the best model) also included the interaction between bug stage and human blood meal, but had no significant effect in the averaged model (Table in S4 Table). Slope coefficients were significant in all other cases (Table in S4 Table). On a post-hoc basis we verified that the successive addition of the number of resident humans, the presence of indoor-nesting chickens (a binary variable) and bug density per site (including squared bug density) did not improve the fit of the model.

### Female fecundity

Mean female fecundity (chorionated eggs per female) was maximal in chicken-fed bugs from chicken coops (15.1), which also had maximal blood-feeding rates [23]; submaximal both in human-fed bugs from domiciles and in pig-fed bugs from pig corrals (13.3), and minimal in goat-fed bugs from goat (7.3) or pig (8.0) corrals (Table 4). The mean fecundity of chicken-fed females in kitchens (12.5) and storerooms (10.9) was intermediate. Using negative binomial regression, we inferred that fecundity increased highly significantly with host blood, showing reduced fecundity in goat- and cat-fed females relative to human-fed females; weakly significantly with log L_c_, and was not modified by a recent feeding (Table in S5 Table, model 5).

**Table 4:** Chorionated eggs per individual female of *T*. *infestans* according to her host-feeding source and habitat. Cat^a^ and mixed blood meals are excluded. Figueroa, October 2003 (austral spring).

	Female fecundity by host blood source														
	Human			Chicken			Dog			Goat			Pig		
Habitat	Mean	SE	N	Mean	SE	N	Mean	SE	N	Mean	SE	N	Mean	SE	N
Domicile	13.3	1.1	49	8.5	2.1	11	6.3	6.3	3			0	18.0		1
Chicken coop			0	15.1	1.8	23			0			0			0
Kitchen			0	12.5	1.8	23			0			0	33		1
Storeroom	7.0		1	10.9	1.5	20	3.5	3.5	2	2.5	2.5	2			0
Goat corral			0			0			0	7.3	3.4	18	10.3	2.4	6
Pig corral			0	0		11			0	8.0	2.0	2	13.3	2.1	21
Total	13.2	1.1	50	12.3	0.9	77	5.2	3.7	5	6.9	2.8	22	13.6	1.8	29

^a^ Mean fecundity for 9 females fed only on cats was 3.9 ± 1.9 eggs.

In human sleeping quarters, Table 4 shows that human-fed females had a 47–64% greater mean number of chorionated eggs per bug (13.3) than chicken- (8.5) or dog-fed bugs (6.3), consistent with human-fed domestic bugs having the largest BMC (Table 2). The fecundity of domestic females increased highly significantly with increasing log L_c_ (P = 0.02), a recent feeding (P < 0.002), having an unmixed human blood meal (P = 0.003) rather than an unmixed feeding on chicken or other host, and the interaction between an unmixed human blood meal and a recent feeding (Table in S5 Table, model 6).

Additional analyses did not reveal significant effects of local host and domestic bug abundance on log BMC and female fecundity, probably because of the rather limited range of variation of bug abundance 2–3 years after an insecticide spraying campaign combined with the relative imprecision of timed-manual bug collections and assessments of local host availability (see below).

## Discussion

Our study is the first to reveal allometric effects of body size surrogates (L and net W) on individual BMC and female fecundity in a large set of triatomine populations, and to show that human- and chicken-fed bugs with unmixed blood meals had significantly larger BMC and fecundity than bugs fed on other hosts only. These novel results in Triatominae, in line with evolutionary theory [7] and partially with the available experimental evidence [13], further strengthen the view that chicken coops and human sleeping quarters are high-quality habitats for *T*. *infestans* in spring, when population size increases rapidly. We also provide the first direct estimates of how much host blood is sequestered in triatomine foreguts, which combined with improved estimates of bug population size may be used to compute total host blood loss.

### Host-feeding, bloodmeal contents and fecundity

The larger BMC and fecundity of triatomines fed only on chickens or humans in their respective main habitats matched their greater blood-feeding rates relative to other habitats and host blood sources [23,25]. Because chicken blood is less nutritious and costlier metabolically than mammalian blood, though less viscous and lacking platelets [5,16], bugs may compensate for increased costs by engorging more easily on chicken blood. Human blood had 2.7 times more protein than chicken blood used to feed sand flies, and twice as much hemoglobin and hematocrit as chicken blood [9]. Rather than indicating defined blood-feeding preferences, host-related BMC differentials and fecundity in domestic bugs most likely reflect the stable availability of an average of 5 human residents versus the seasonally variable presence of 1–2 indoor-nesting chickens (relative host availability) and greater nutritional value of human blood. We infer that domestic and chicken-coop bugs often made partial blood meals because their median blood-feeding intervals (4.1 and 2.8 days, respectively) [23] are a small fraction of the total time required for digesting a full blood meal, and because unmixed blood meals indicate repeated feedings on the same host species [27]. Whether the partial blood meals were generated by the hosts’ defensive reactions or its immune response to salivary antigens is beyond the scope of our study.

Our data do not allow a direct inference on whether human blood provided an advantage over chicken blood, as shown experimentally [13], because (i) human feeding is nearly exclusively associated with domestic habitats, which provide more stable conditions [41] and host availability than most peridomestic structures, and (ii) domestic bugs fed on humans substantially more frequently than on chickens in our study [28] but not elsewhere in Santiago del Estero [27], for example.

Goat-fed populations of *T*. *infestans* ranked last in BMC and fecundity, in close agreement with habitat-specific mean blood-feeding rates and engorgement status [23,25]. The inferiority of goat (or sheep or cow) blood for triatomines is supported by experimental evidence [11,13,14]. Triatomines lack the ability to agglutinate cattle erythrocytes (unlike erythrocytes from mice, rats and rabbits), which reduced the feeding efficiency of *T*. *brasiliensis* [42]. Gardiner and Maddrell [11, p. 513] reported that *R*. *prolixus* tended to blood-feed much less or not at all on adult goats and sheep that had been exposed repeatedly to triatomines, and consequently produced far fewer eggs. As this phenomenon did not occur in goat kids or naïve sheep, they inferred that repeatedly exposed goats and sheep might have developed acquired resistance to triatomine bites after 1–9 weeks of exposure [11]. Likewise, *R*. *prolixus* females fed on restrained mice with little or no previous exposure laid 1.7 times as many eggs as bugs fed similarly on repeatedly-exposed mice [43], but differential host restlessness and lack of bloodmeal size estimates do not allow a definite conclusion. In summary, both digestive and immune processes may account for the reduced ability of triatomines to engorge fully on goats and other bovine hosts depending on their history of exposure to bites.

How may triatomines from goat corrals achieve large population sizes if goats are a less suitable host via acquired resistance? The answer is most likely related to the high recruitment rates of goats and seasonal husbandry practices in semi-arid or arid rural areas of the Gran Chaco. Goat corrals there traditionally have fences made of piled thorny shrubs and goat manure forming a thick matrix where triatomines thrive and which damps the daily extremes of temperature [31,32,44,45]. Goats deliver twice a year and lactating kids are kept almost permanently for 2–3 months in a small thatched enclosure frequently crowded with triatomines [25,45]. Because kids are immunologically naïve, they may allow greater feeding success and bloodmeal size than adult goats with acquired resistance to triatomine bites. Our current results backed by experimental evidence provide an additional, non-exclusive mechanistic explanation of the reduced blood-feeding rate and engorgement of goat-fed bugs recorded elsewhere [25]. Acquired host resistance combined with shifting host availability and harsh environmental conditions would trigger triatomine dispersal and house invasion at appropriate times [23,25,46,47].

Fifth-instar nymphs had larger BMC than other stages, consistent with their larger bloodmeal size [12,22], submaximal post-feeding defecation rates [48], and large rates of infection with *T*. *cruzi* [49]. Fifth instars are key life stages because they also have sub-maximal reproductive value, larger survival when starved or exposed to insecticides [50]. The body size and associated blood-feeding of fifth instars determine adult body size and hence affect several fitness components.

### Body size

Body length L correlated closely with other metrics of body size (total W and net W) for most bug stages and was associated positively with nymphal BMC and domestic female fecundity, as predicted by evolutionary theory and recorded in numerous insect species other than triatomines [18]. Triatomines from chicken coops (followed by domestic bugs) were significantly larger than bugs from other habitats across most life stages–a clear sign of combined habitat- and host-associated fitness advantages. Similarly, in the dry Argentine Chaco, *T*. *infestans* adults collected in domiciles and chicken coops had consistently larger wing centroid size (which correlated positively and highly significantly with L) than bugs collected from goat or pig corrals and wood piles in Santiago del Estero [51], whereas fifth instars and adults from chicken coops had bigger wing and head centroid sizes than goat-corral triatomines in La Rioja [52]. This pattern was not recorded in the humid Chaco [53,54], suggesting the occurrence of other sources of variation not accounted for. Among them, major differences between regions in the physical structure and construction materials used in domiciles, chicken coops and corrals (affecting their capacity to damp climatic extremes) and the well-known xeropreference of *T*. *infestans* may exert important effects on body size [8].

Insect body size is subject to considerable plastic variation related to nutrition, temperature and other environmental factors, and genes [55]. Assuming that relevant genetic effects affecting insect growth rate [26,55] would average out among bug subpopulations at this limited spatial scale, the recorded body-size differentials may be attributed to improved nutrition (determined by host blood quality, host density and feeding frequency) and local temperature regimes. A history of large BMC at each nymphal instar combined with high blood-feeding frequencies imply near-maximal proportional growth ratios between successive instars (Dyar’s rule [55]), with growth extending even after attaining the critical weight to molt, leading to larger adult body sizes. This chain of events may be sufficient to explain the trajectory of higher L across most bug stages in chicken coops and the smaller size of goat-corral *T*. *infestans*.

Insect body size is affected by habitat-specific microsite temperatures [56]. Triatomine species display clear patterns of thermopreference and hygropreference, which vary substantially over the feeding cycle and affect refuge selection [8]. The main habitats of *T*. *infestans* largely differed in average microsite temperatures and relative humidity, as did their capacity to damp external temperatures, ranging from minimal in goat corrals to maximal in mud-and-thatch domiciles [41]. Fluctuating temperature regimes with large thermal amplitudes reduce insect body size and female fecundity when they encompass stressful temperatures [56]. Development times affect the growth rates of body size and also depend on the amplitude of temperature variations and whether they exceed the heat-injury zone [56]. In goat corrals, where daily swings of 12–15°C across seasons and maxima frequently exceeded 40°C over spring-summer [41], these variations most likely reduced adult body size and caused the largest levels of fluctuating asymmetry observed in the wing shape of the study female *T*. *infestans* across habitats [57]–another indication of developmental stress [56].

### Limitations

Our study in early spring provides an instantaneous representation of a seasonally variable environment in which the current status of bug populations depends on past, unknown conditions. Because of the 5–7 month-long development period, most triatomines collected in early spring probably emerged by late summer and mid fall. Our measures of the number of chorionated eggs (fecundity) and reported local host abundance (host occupancy) are point approximations, whereas host-feeding patterns reflect prevailing conditions of host availability over the previous 1–3 months [27]. These unrecorded variations hamper assessing the relation between BMC and local host abundance. Chorionated oocytes represent a terminal event in oogenesis and stay in the terminal oviduct for a short fraction of the total time required for egg development.

The standing size distributions of field triatomine populations are potentially affected by unknown in-migration and out-migration rates. For example, *T*. *infestans* males typically display large mobility and propensity to initiate flight [23,25,58] and their phenotypes may more frequently correspond to habitats other than in those they were collected, which introduces a source of error.

Other limitations of our study are that L and other related attributes lack estimates of measurement error, and that information on microsite temperature and other conditions were borrowed from a related study within the same region [25,41]. Although the effects of human and chicken hosts on triatomine life-history traits appear to be generalized, regional differences in habitat types, domestic hosts’ exposure, animal management and triatomine species preclude sweeping generalizations.

### Implication for vector control and population dynamics

Our study discloses the pervasive effects of body size on various fitness components that affect the risks of transmission of *T*. *cruzi*. Greater L entails greater BMC and hence greater risks of acquiring an infection and of emitting dejecta during or shortly after feeding [20,21], potentially leading to contamination of hosts' skin with trypanosomes. L was also closely and positively correlated with *T*. *infestans* female fecundity, wing centroid size [53], and flight initiation probabilities [58,59]. Larger females had larger wing muscle mass and greater flight initiation rates than males and smaller females [60], and hence would have a greater dispersal range and resistance to starvation.

Our study also discloses the triple role of goat (and possibly sheep) corrals, which may function as productive sites (during the breeding season when kids are enclosed); as sources of dispersants (when naïve kids or adult goats are few or unavailable and high temperatures or other harsh conditions stimulate flight initiation), and as refuges where bugs are more likely to survive residual spraying with insecticides [31,47]. Chicken-coop and domestic triatomine populations displayed near-maximal values in virtually every life-history trait examined. Such large effects on various fitness components impact bug population dynamics and are relevant for designing improved vector control strategies with intensified actions on high-quality habitats.

## Supporting information

S1 Fig Sample flow chart from field bug collection through testing of bloodmeal contents. Figueroa, October 2003 (austral spring).
(TIF)

S2 Fig Frequency distribution of log bloodmeal contents (mg) (a), log body length (mm) (b), log total body weight (mg) (c), and log net weight (mg) (d) in *T*. *infestans* collected in domestic and peridomestic habitats. Figueroa, October 2003 (austral spring).
(TIF)

S3 Fig Mean log body length (mm) by bug stage (plus or minus one standard error) according to having a recent feeding or not in chicken coops (a), domiciles (b), kitchens (c), pig corrals (d) and storerooms (e) in *T*. *infestans* collected in domestic and peridomestic habitats. Figueroa, October 2003 (austral spring).
(TIF)

S1 Table Individual insect data including infestation and stage-specific bug abundance per site; recent blood-feeding, nutritional and engorgement status; bloodmeal contents and identification results, and body size of (peri)domestic *T*. *infestans*. Figueroa, October 2003 (austral spring). (XLS)

S2 Table Ordinary least-square regression of log bloodmeal contents (mg) on mean-centered log body length (mm) or log weight:length (mg/mm) or log net body weight (mg), and log net body weight on log centered body length, by bug stage. Figueroa, October 2003 (austral spring).
(DOCX)

S3 Table Random-intercept multiple linear regression model of log length (mm) (the response variable) on bug habitat, stage, and a recent feeding in *T*. *infestans* collected in (peri)domestic habitats. Figueroa, October 2003 (austral spring).
(DOCX)

S4 Table Random-intercept multiple linear regression models of log bloodmeal contents (mg) (the response variable) on mean-centered log length (L_c_), bug stage, and a recent feeding and bug habitat (model 2) or host blood source (model 3) or human blood meal from domiciles (model 4) in *T*. *infestans* collected in domestic and peridomestic habitats. Figueroa, October 2003 (austral spring).
(DOCX)

S5 Table Negative binomial regression clustered by collection site of the number of chorionated eggs per female on having an unmixed human blood meal (as opposed to feeding on chicken or on other hosts only), female mean-centered log length (L_c_), and a recent feeding in *T*. *infestans* collected in all habitats (model 5) or in domestic habitats (model 6). Figueroa, October 2003 (austral spring).
(DOCX)

S1 Text A brief review of whether feeding on mammals (including humans) confers feeding and fitness advantages compared to feeding on birds. (DOCX)
