# Supplementary figures and images for: Body size and hosts of Triatoma infestans populations affect the size of bloodmeal contents and female fecundity in rural northwestern Argentina
Source: PLoS Negl Trop Dis. 2017 Dec 6;11(12):e0006097. doi: 10.1371/journal.pntd.0006097 (PMC5734792; doi:10.1371/journal.pntd.0006097)

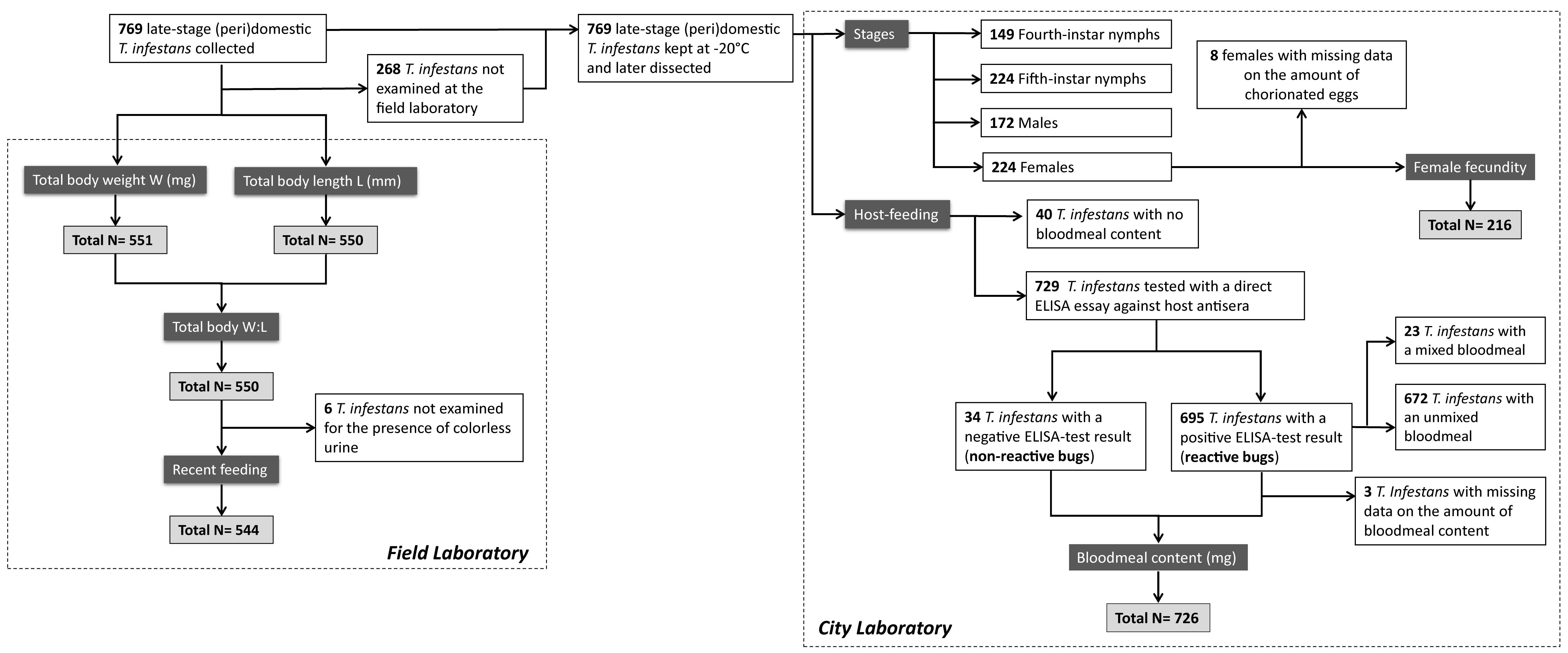

Supplement: S1 Fig — Figueroa, October 2003 (austral spring). (TIF) [file pntd.0006097.s001.tif]

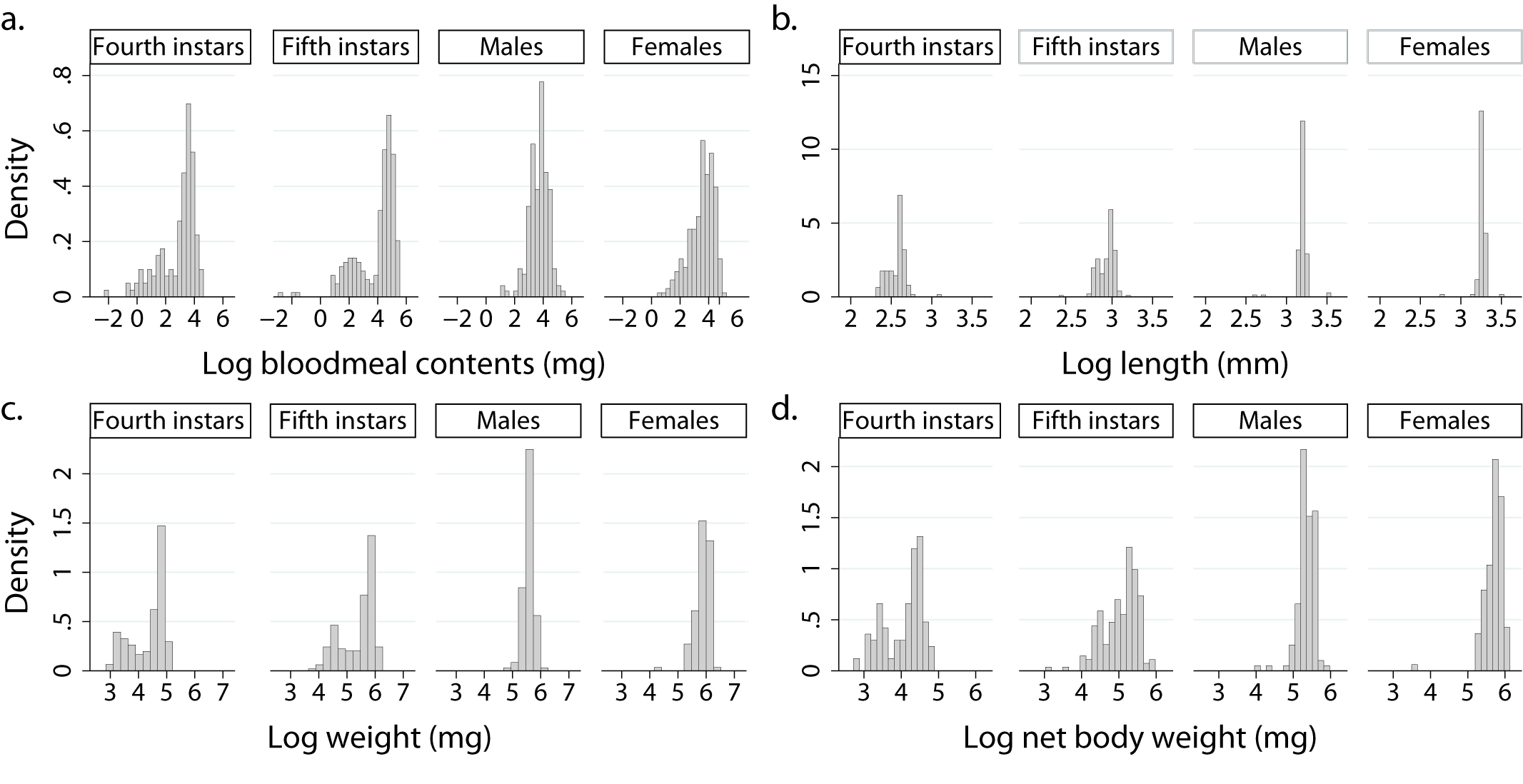

Supplement: S2 Fig — Frequency distribution of log bloodmeal contents (mg) (a), log body length (mm) (b), log total body weight (mg) (c), and log net weight (mg) (d) in T. infestans collected in domestic and peridomestic habitats. Figueroa, October 2003 (austral spring). (TIF) [file pntd.0006097.s002.tif]

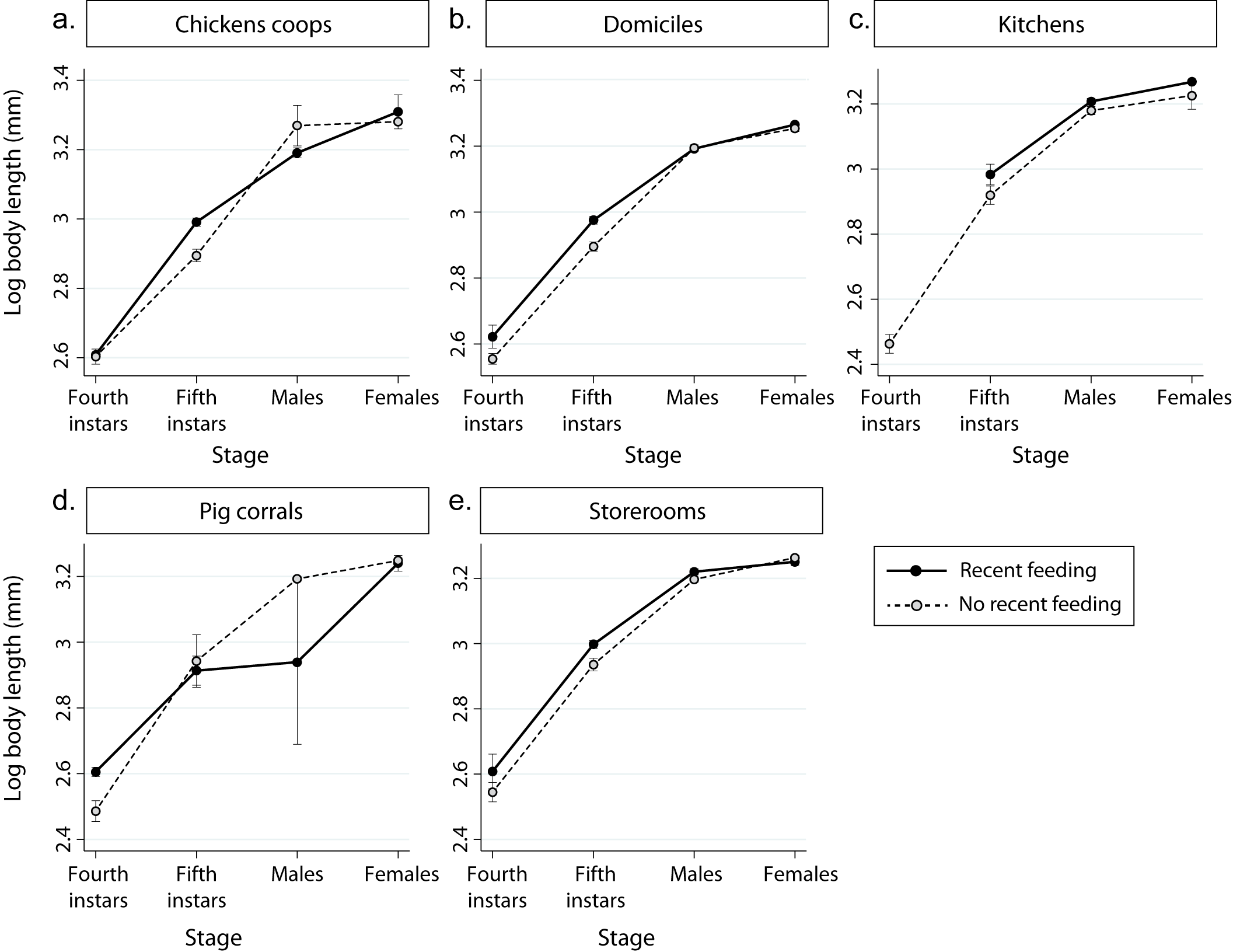

Supplement: S3 Fig — Mean log body length (mm) by bug stage (plus or minus one standard error) according to having a recent feeding or not in chicken coops (a), domiciles (b), kitchens (c), pig corrals (d) and storerooms (e) in T. infestans collected in domestic and peridomestic habitats. Figueroa, October 2003 (austral spring). (TIF) [file pntd.0006097.s003.tif]
