# Supplementary material for: Body size and hosts of Triatoma infestans populations affect the size of bloodmeal contents and female fecundity in rural northwestern Argentina
Source: PLoS Negl Trop Dis. 2017 Dec 6;11(12):e0006097. doi: 10.1371/journal.pntd.0006097 (PMC5734792; doi:10.1371/journal.pntd.0006097)
Supplement: S2 Table — Figueroa, October 2003 (austral spring). (DOCX) [file pntd.0006097.s005.docx]

**S2 Table.** Ordinary least-square regression of log bloodmeal contents (mg) on mean-centered log body length (mm) or log weight:length (mg/mm) or log net body weight (mg), and log net body weight on log centered body length, by bug stage. Figueroa, October 2003 (austral spring).

| Response | Explanatory |  |  |  |  |  |  |  |
| --- | --- | --- | --- | --- | --- | --- | --- | --- |
| variable | variable | Stage | Intercept a | SE a | Coefficient b | SE b | Adj. R^2^ | df |
| Log BMC | Log L_c_ | Fourth instar | 2.8458 | 0.1020 | 6.9060 | 0.9513 | 0.324 | 107 |
|  |  | Fifth instar | 3.9593 | 0.0620 | 10.3533 | 0.6306 | 0.604 | 175 |
|  |  | Female | 3.8739 | 0.0702 | 0.0760 | 1.1728 | -0.010 | 104 |
|  |  | Male | 3.8303 | 0.0563 | -0.1417 | 0.6592 | -0.008 | 175 |
|  |  |  |  |  |  |  |  |  |
| Log BMC | Log W:L | Fourth instar | -0.4499 | 0.2746 | 1.8472 | 0.1446 | 0.600 | 107 |
|  |  | Fifth instar | -1.8059 | 0.2435 | 2.3254 | 0.0958 | 0.771 | 174 |
|  |  | Female | -0.8440 | 0.6202 | 1.8120 | 0.2372 | 0.353 | 104 |
|  |  | Male | -0.9155 | 0.5698 | 1.9896 | 0.2381 | 0.355 | 124 |
|  |  |  |  |  |  |  |  |  |
| Log BMC | Log net weight | Fourth instar | -3.4337 | 0.7851 | 1.5552 | 0.1914 | 0.380 | 105 |
|  |  | Fifth instar | -5.0459 | 0.7994 | 1.7919 | 0.1578 | 0.422 | 174 |
|  |  | Female | 0.7346 | 1.3342 | 0.5522 | 0.2344 | 0.042 | 104 |
|  |  | Male | 5.2400 | 1.3094 | -0.2640 | 0.2447 | 0.001 | 125 |
|  |  |  |  |  |  |  |  |  |
| Log net weight | Log L_c_ | Fourth instar | 4.0354 | 0.0409 | 2.8576 | 0.3800 | 0.342 | 106 |
|  |  | Fifth instar | 5.0277 | 0.0219 | 3.8469 | 0.2213 | 0.634 | 173 |
|  |  | Female | 5.6839 | 0.0223 | 3.0533 | 0.3728 | 0.386 | 104 |
|  |  | Male | 5.3464 | 0.0202 | 0.3414 | 0.2371 | 0.008 | 125 |
