# Supplementary material for: Body size and hosts of Triatoma infestans populations affect the size of bloodmeal contents and female fecundity in rural northwestern Argentina
Source: PLoS Negl Trop Dis. 2017 Dec 6;11(12):e0006097. doi: 10.1371/journal.pntd.0006097 (PMC5734792; doi:10.1371/journal.pntd.0006097)
Supplement: S3 Table — Figueroa, October 2003 (austral spring). (DOCX) [file pntd.0006097.s006.docx]

**S3** **Table.** Random-intercept multiple linear regression model of log length (mm) (the response variable) on bug habitat, stage, and a recent feeding in *T. infestans* collected in (peri)domestic habitats. Full model average of models with an evidence ratio >0. Reference levels were fourth instars, and no recent feeding, respectively. Figueroa, October 2003 (austral spring).

| Model set | Explanatory variable | Levels in model | Coefficient β | SE β | P | RI |
| --- | --- | --- | --- | --- | --- | --- |
| 1^a^ | Stage | Fifth instars | 0.3570 | 0.0125 | <0.001 | 1 |
| 1 |  | Males | 0.6529 | 0.0132 | <0.001 |  |
| 1 |  | Females | 0.7071 | 0.0147 | <0.001 |  |
| 1 | Recent feeding | Yes | 0.0643 | 0.0195 | <0.001 | 1 |
| 1 | Stage*recent feeding | Fifth instars* recent feeding (yes) | 0.0001 | 0.0065 | >0.1 | 1 |
| 1 |  | Males* recent feeding (yes) | -0.0071 | 0.0253 | <0.001 |  |
| 1 |  | Females* recent feeding (yes) | -0.0041 | 0.0157 | 0.03 |  |
| 1 | Habitat | Pig Corral | -0.0058 | 0.0169 | >0.1 | 0.3 |
| 1 |  | Storeroom | 0.0068 | 0.0159 | >0.1 |  |
| 1 |  | Domicile | 0.0035 | 0.0114 | >0.1 |  |
| 1 |  | Chicken coop | 0.0107 | 0.0197 | >0.1 |  |
| 1 | Habitat*recent feeding | Pig Corral* recent feeding (yes) | -0.0029 | 0.0174 | >0.1 | 0.04 |
| 1 |  | Storeroom* recent feeding (yes) | -0.0006 | 0.0080 | >0.1 |  |
| 1 |  | Domicile* recent feeding (yes) | -0.0005 | 0.0066 | >0.1 |  |
| 1 |  | Chicken coop* recent feeding (yes) | -0.0009 | 0.0087 | >0.1 |  |
| 1 | Intercept |  | 2.5437 | 0.0151 | <0.001 |  |
| 1 | ^d^ √ψ |  | 0.0147 |  |  |  |
| 1 | ^e^ √θ |  | 0.0858 |  |  |  |
| RI= relative importance of the variable or interaction | | | | | | |
| ^a^ Likelihood-ratio test of √ψ = 0: χ^2^ (1 df) = 1.67; P = 0.2 | | | | | | |
| ^d^ √ψ between-cluster standard deviation | | | | | | |
| ^e^ √θ within-cluster standard deviation | | | | | | |
