# Supplementary material for: Body size and hosts of Triatoma infestans populations affect the size of bloodmeal contents and female fecundity in rural northwestern Argentina
Source: PLoS Negl Trop Dis. 2017 Dec 6;11(12):e0006097. doi: 10.1371/journal.pntd.0006097 (PMC5734792; doi:10.1371/journal.pntd.0006097)
Supplement: S4 Table — Figueroa, October 2003 (austral spring). (DOCX) [file pntd.0006097.s007.docx]

**S4 Table.** Random-intercept multiple linear regression models of log bloodmeal contents (mg) (the response variable) on mean-centered log length (L_c_), bug stage, and a recent feeding and bug habitat (model 2) or host blood source (model 3) or human blood meal from domiciles (model 4) in *T. infestans* collected in domestic and peridomestic habitats. Full model average of models with an evidence ratio >0. Figueroa, October 2003 (austral spring).

| Model set | Explanatory variable | Levels in model | Coefficient β | SE β | P | RI |
| --- | --- | --- | --- | --- | --- | --- |
| 2^a^ | Habitat | Pig corral | 0.3936 | 0.2817 | >0.1 | 0.9 |
| 2 |  | Storeroom | 0.3014 | 0.2579 | >0.1 |  |
| 2 |  | Domicile | 0.3678 | 0.2173 | 0.09 |  |
| 2 |  | Chicken coop | 0.3180 | 0.2415 | >0.1 |  |
| 2 | Recent feeding | Yes | 0.5854 | 0.1093 | <0.001 | 1 |
| 2 | Stage | Fifth instars | 0.9997 | 0.0930 | <0.001 | 1 |
| 2 |  | Males | 0.8401 | 0.1012 | <0.001 |  |
| 2 |  | Females | 0.7986 | 0.1067 | <0.001 |  |
| 2 | L_c_ | Mean-centered body length | 7.7593 | 1.5485 | <0.001 | 1 |
| 2 | Habitat*L_c_ | Pig corral*L_c_ | 4.5026 | 2.3352 | 0.05 | 0.9 |
| 2 |  | Storeroom*L_c_ | 1.2427 | 1.7888 | >0.1 |  |
| 2 |  | Domicile*L_c_ | -0.7449 | 1.5144 | >0.1 |  |
| 2 |  | Chicken coop*L_c_ | 2.1870 | 1.7798 | >0.1 |  |
| 2 | Recent feeding*L_c_ | Yes*L_c_ | -4.3129 | 0.9324 | <0.001 | 1 |
| 2 | Stage*L_c_ | Fifth instars*L_c_ | 2.3482 | 0.8846 | <0.001 | 1 |
| 2 |  | Males*L_c_ | -7.8500 | 1.1607 | <0.001 |  |
| 2 |  | Females*L_c_ | -7.4398 | 1.3629 | <0.001 |  |
| 2 | Habitat*Recent feeding | Pig corral*Yes | -0.0235 | 0.1393 | >0.1 | 0.04 |
| 2 |  | Storeroom*Yes | -0.0174 | 0.1062 | >0.1 |  |
| 2 |  | Domicile*Yes | -0.0127 | 0.0826 | >0.1 |  |
| 2 |  | Chicken coop*Yes | -0.0110 | 0.0838 | >0.1 |  |
| 2 | Intercept |  | 2.5095 | 0.2190 | <0.001 |  |
| 2 | ^d^ √ψ |  | 0.2208 |  |  |  |
| 2 | ^e^ √θ |  | 0.7011 |  |  |  |
|  |  |  |  |  |  |  |
| 3^b^ | Recent feeding | Yes | 0.5120 | 0.1022 | <0.001 | 1 |
| 3 | Stage | Fifth instars | 1.0090 | 0.0965 | <0.001 | 1 |
| 3 |  | Males | 0.8632 | 0.1001 | <0.001 |  |
| 3 |  | Females | 0.8298 | 0.1064 | <0.001 |  |
| 3 | L_c_ | Mean-centered body length | 5.9290 | 0.8815 | <0.001 | 1 |
| 3 | Host blood meal | Chicken | -0.2386 | 0.0839 | 0.004 | 1 |
| 3 |  | Dog | -0.0512 | 0.1643 | >0.1 |  |
| 3 |  | Cat | -1.1290 | 1.0850 | >0.1 |  |
| 3 |  | Goat | -0.0755 | 0.2021 | >0.1 |  |
| 3 |  | Pig | -0.2039 | 0.1917 | >0.1 |  |
| 3 | Stage*L_c_ | Fifth instars*L_c_ | 1.5480 | 0.8599 | 0.07 | 1 |
| 3 |  | Males*L_c_ | -7.5070 | 0.9735 | <0.001 |  |
| 3 |  | Females*L_c_ | -9.0600 | 1.2310 | <0.001 |  |
| 3 | Host blood*L_c_ | Chicken*L_c_ | 3.9820 | 0.7704 | <0.001 | 1 |
| 3 |  | Dog*L_c_ | 3.6160 | 1.7420 | 0.04 |  |
| 3 |  | Cat*L_c_ | -48.6900 | 62.2500 | >0.1 |  |
| 3 |  | Goat*L_c_ | 0.0001 | 2.7210 | >0.1 |  |
| 3 |  | Pig*L_c_ | 6.4920 | 1.2840 | <0.001 |  |
| 3 | Recent feeding*L_c_ | Yes*L_c_ | -4.4240 | 0.8785 | <0.001 | 1 |
| 3 | Stage*Recent feeding | Fifth instars*Yes | 0.0924 | 0.1597 | >0.1 | 0.04 |
| 3 |  | Males*Yes | -0.0661 | 0.1482 | >0.1 |  |
| 3 |  | Females*Yes | -0.0053 | 0.1178 | >0.1 |  |
| 3 | Stage*Host blood | Chicken* Fifth instars | -0.0008 | 0.0220 | >0.1 | 0.01 |
| 3 |  | Dog* Fifth instars | 0.0012 | 0.0471 | >0.1 |  |
| 3 |  | Cat* Fifth instars | - | - | - |  |
| 3 |  | Goat* Fifth instars | 0.0016 | 0.0543 | >0.1 |  |
| 3 |  | Pig* Fifth instars | 0.0155 | 0.1429 | >0.1 |  |
| 3 |  | Chicken* Males | 0.0026 | 0.0318 | >0.1 |  |
| 3 |  | Dog* Males | 0.0043 | 0.0671 | >0.1 |  |
| 3 |  | Cat* Males | 0.0000 | 0.0000 | >0.1 |  |
| 3 |  | Goat* Males | 0.0036 | 0.0633 | >0.1 |  |
| 3 |  | Pig* Males | 0.0153 | 0.1401 | >0.1 |  |
| 3 |  | Chicken* Females | 0.0025 | 0.0317 | >0.1 |  |
| 3 |  | Dog* Females | 0.0057 | 0.0749 | >0.1 |  |
| 3 |  | Cat* Females | 3.2*10^-10^ | 2.5*10^-5^ | >0.1 |  |
| 3 |  | Goat* Females | 0.0034 | 0.0604 | >0.1 |  |
| 3 |  | Pig* Females | 0.0142 | 0.1332 | >0.1 |  |
| 3 | Intercept |  | 3.0200 | 0.0934 | <0.001 |  |
| 3 | ψ |  | 0.1711 |  |  |  |
| 3 | θ |  | 0.5905 |  |  |  |
|  |  |  |  |  |  |  |
| 4^c^ | Recent feeding | Yes | 0.4577 | 0.0923 | <0.001 | 1 |
| 4 | Stage | Fifth instars | 0.9871 | 0.1733 | <0.001 | 1 |
| 4 |  | Males | 0.8336 | 0.1915 | <0.001 |  |
| 4 |  | Females | 0.7508 | 0.1807 | <0.001 |  |
| 4 | L_c_ | Mean-centered body length | 10.5856 | 1.3355 | <0.001 | 1 |
| 4 | Human blood | Human | 0.3729 | 0.1901 | 0.05 | 1 |
| 4 |  | Other blood source | -0.4322 | 0.3179 | >0.1 |  |
| 4 | Stage*L_c_ | Fifth instars*L_c_ | 2.2592 | 1.2127 | 0.06 | 1 |
| 4 |  | Males*L_c_ | -5.2861 | 1.6720 | 0.001 |  |
| 4 |  | Females*L_c_ | -10.9087 | 1.7427 | <0.001 |  |
| 4 | Human blood*L_c_ | Human*L_c_ | -5.9007 | 1.4453 | <0.001 | 1 |
| 4 |  | Other blood source*L_c_ | -6.4902 | 1.7783 | <0.001 |  |
| 4 | Recent feeding*L_c_ | Yes*L_c_ | -3.1547 | 1.4617 | 0.03 | 0.9 |
| 4 | Human blood*Stage | Human*Fifth instars | -0.0085 | 0.1518 | >0.1 | 0.3 |
| 4 |  | Human*Males | -0.1396 | 0.2638 | >0.1 |  |
| 4 |  | Human*Females | -0.0844 | 0.2087 | >0.1 |  |
| 4 |  | Other blood source*Fifth instars | 0.2660 | 0.4540 | >0.1 |  |
| 4 |  | Other blood source*Males | 0.2078 | 0.3919 | >0.1 |  |
| 4 |  | Other blood source*Females | 0.2042 | 0.4030 | >0.1 |  |
| 4 | Intercept |  | 2.7931 | 0.1511 | <0.001 |  |
| 4 | √ψ ^d^ |  | 0.2009 |  |  |  |
| 4 | √θ ^e^ |  | 0.6054 |  |  |  |
| RI= relative importance of the variable or interaction | | | | | | |
| ^a^ Likelihood-ratio (LR) test of √ψ = 0: χ2 (1 df) = 22.1, P < 0.001 | | | | | | |
| ^b^ Likelihood-ratio test of √ψ = 0: χ2 (1 df) = 14.83, P < 0.001 | | | | | | |
| ^c^ Likelihood-ratio test of √ψ = 0: χ2 (1 df) = 4.42, P = 0.03 | | | | | | |
| ^d^ √ψ between-cluster standard deviation | | | | | | |
| ^e^ √θ within-cluster standard deviation | | | | | | |
