# Supplementary material for: Body size and hosts of Triatoma infestans populations affect the size of bloodmeal contents and female fecundity in rural northwestern Argentina
Source: PLoS Negl Trop Dis. 2017 Dec 6;11(12):e0006097. doi: 10.1371/journal.pntd.0006097 (PMC5734792; doi:10.1371/journal.pntd.0006097)
Supplement: S5 Table — Figueroa, October 2003 (austral spring). (DOCX) [file pntd.0006097.s008.docx]

**S5 Table.** Negative binomial regression clustered by collection site of the number of chorionated eggs per female on having an unmixed human blood meal (as opposed to feeding on chicken or on other hosts only), female mean-centered log length (L_c_), and a recent feeding in *T. infestans* collected in all habitats (model 5) or in domestic habitats (model 6). Figueroa, October 2003 (austral spring).

|  | Explanatory | Levels in |  |  |  |
| --- | --- | --- | --- | --- | --- |
| Model set | Variable | model | Coefficient b | SE b | P |
| 5. All | Recent feeding | Yes | -0.1987 | 0.2222 | >0.1 |
| habitats | L_c_ | Mean-centered body length | 1.7270 | 1.0455 | 0.099 |
| 5 | Host blood meal | Chicken | -0.3678 | 0.2317 | >0.1 |
| 5 |  | Dog | -1.1284 | 0.8856 | >0.1 |
| 5 |  | Cat | -1.2950 | 0.1135 | <0.001 |
| 5 |  | Goat | -1.7591 | 0.1125 | <0.001 |
| 5 |  | Pig | 0.2920 | 0.1387 | 0.035 |
| 5 | Host blood*Recent feeding | Chicken*Yes | 0.5497 | 0.3011 | 0.068 |
| 5 |  | Goat*Yes | 1.0774 | 0.2226 | <0.001 |
| 5 |  | Pig*Yes | -0.7370 | 0.2449 | 0.003 |
| 5 | Constant |  | 2.6663 | 0.1113 | <0.001 |
| 5 | α ^a^ |  | 0.4853 | 0.1318 |  |
| 6. Domestic | Recent feeding | Yes | 1.0690 | 0.3476 | 0.002 |
| habitats | L_c_ | Mean-centered body length | 6.9158 | 2.9771 | 0.02 |
| 6 | Human blood | Human | 1.0874 | 0.3709 | 0.003 |
| 6 |  | Other blood source | 0.1080 | 0.7314 | >0.1 |
| 6 | Human blood *Recent feeding | Human*Yes | -1.3121 | 0.4429 | 0.003 |
| 6 | Constant |  | 1.5765 | 0.3466 | <0.001 |
| 6 | α ^a^ |  | 0.5840 | 0.1663 |  |

^a^ α variance of the exponential of random intercept
