# Supplementary material for: Body size and hosts of Triatoma infestans populations affect the size of bloodmeal contents and female fecundity in rural northwestern Argentina
Source: PLoS Negl Trop Dis. 2017 Dec 6;11(12):e0006097. doi: 10.1371/journal.pntd.0006097 (PMC5734792; doi:10.1371/journal.pntd.0006097)
Supplement: S1 Text — (DOCX) [file pntd.0006097.s009.docx]

Body size and hosts of *Triatoma infestans* populations affect the size of bloodmeal contents and female fecundity in rural northwestern Argentina

Ricardo E. Gürtler, María del Pilar Fernández, María Carla Cecere, Joel E. Cohen

Supplementary Text 1

A brief review of whether feeding on mammals (including humans) confers feeding and fitness advantages compared to feeding on birds

Early studies on mass rearing of triatomines reported that the type of host blood affected the insects’ vital rates and engorgement levels [1-3]. *Rhodnius prolixus* bugs fed artificially on defribinated blood through a membrane achieved greater adult size and egg weight after feeding on pig rather than on cow blood during two generations [4]. Human blood was better than sheep blood for vitellogenesis, general metabolism and development in *R. prolixus* [5]. Cohorts of *R. prolixus* fed artificially on citrated human and rabbit blood through a membrane during their entire lives achieved faster development and much larger bloodmeal size, body weight and female fecundity than bugs fed similarly on chicken, sheep and horse blood [6]. In contrast, *R. prolixus* females experimentally fed twice on immobilized pigeons and chickens rather than on citrated human blood differed little in the total quantity of blood ingested whereas female fecundity decreased from pigeons to humans to chickens [7]. *Triatoma brasiliensis* achieved a better feeding performance on human volunteers than on a sedated wild rodent host (*Trichomys laurentius*) to which this vector is typically associated [8].

Several experimental studies assessed whether a mammalian and an avian host differentially affected selected fitness components of triatomines. Fifth-instar nymphs of *T. infestans* feeding on immobilized pigeons achieved substantially greater host-contact times, blood ingestion rates and intake than when feeding on sedated mice, which interrupted blood-feeding more frequently [9]. Cohorts of mouse-fed *T. infestans* had shorter nymphal development times and greater survival, adult body length and hatching success than pigeon-fed bugs, which had consistently greater female fecundity and survival [10]. Similarly, *T. infestans* and *Triatoma patagonica* fed on guinea pigs kept in a small plastic cage achieved higher female fecundity rates and efficiency of conversion of host blood into eggs than bugs fed on immobilized pigeons, which achieved greater blood intake and female life expectancy [11,12]. None of these studies assessed whether the blood from the study pigeons, chickens, mice, guinea pigs and humans differed in nutritional quality.

Although in most of these studies mammalian blood appeared to be more advantageous to bugs' fitness than avian blood, the interpretation of these findings and search for common patterns are hampered by technical and analytical details. Artificial feeding with defibrinated, citrate- or heparin-treated blood may exert adverse effects on the insects depending on the host and triatomine species tested and the anticoagulant dose [2,3,7,13]. Núñez and Segura [3] suggested that *T. infestans* (but not *R. prolixus*) is extremely sensitive to some deleterious substances contained in the artificial food used to feed rabbits and chickens, and that sheep blood (used successfully for *R. prolixus*) is inadequate for *T. infestans*. Because live hosts usually display host species-specific or age-dependent defensive behaviors that affect blood intake, using sedated and immobilized hosts under undefined levels of restraint or immobility introduces another important source of variation. The aggregate fitness implications of host blood type for defined triatomine species in terms of a summary measure of fitness such as the finite rate of population increase remain to be established.

**References**

1. [Friend WG](http://www.ncbi.nlm.nih.gov/pubmed?term=Friend%20WG%5BAuthor%5D&cauthor=true&cauthor_uid=319741), [Smith JJ](http://www.ncbi.nlm.nih.gov/pubmed?term=Smith%20JJ%5BAuthor%5D&cauthor=true&cauthor_uid=319741) (1977) Factors affecting feeding by bloodsucking insects. [Annu Rev Entomol](http://www.ncbi.nlm.nih.gov/pubmed/?term=friend+smith+triatoma+rhodnius) 22: 309-331.

2. Gardiner BO, Maddrell SH (1972) Techniques for routine and large-scale rearing of *Rhodnius prolixus* Stål (Hem., Reduviidae). Bull Entomol Res 61: 505–515.

3. Núñez JA, Segura EL (1987) Rearing of Triatominae. Chagas’ Disease Vectors, Vol. II (eds RR Brenner, AM Stoka), pp. 31–40. Florida: CRC Press.

4. Langley PA, Pimley RW (1978) Rearing triatomine bugs in the absence of a live host and some effects of diet on reproduction in *Rhodnius prolixus* Stål (Hemiptera: Reduviidae). Bull ent Res 68: 243–250.

5. Valle D, Gomes JEPL, Goldenberg S, Garcia ES (1987) *Rhodnius prolixus* vitellogenesis: dependence upon the blood source. J Insect Physiol 33: 249–254.

6. Gomes JE, Azambuja P, Garcia ES (1990) Comparative studies on the growth and reproductive performances of *Rhodnius prolixus* reared on different blood sources. Mem Inst Oswaldo Cruz 85: 299–304.

7. Aldana E, Jácome D, Lizano E (2009) Efecto de la alternación de fuentes sanguíneas sobre la fecundidad y la fertilidad de *Rhodnius prolixus* Stål (Heteroptera: Reduviidae). Entomo Brasilis 2: 17–23.

8. Guarneri AA, Araujo RN, Diotaiuti L, Gontijo NF, Pereira MH (2011) Feeding performance of *Triatoma brasiliensis* (Hemiptera: Reduviidae) on habitual hosts: *Thrichomys laurentius* (Rodentia: Echimyidae) and humans. Vector Borne Zoonotic Dis 11: 443–445.

9. Guarneri AA, Diotaiuti L, Gontijo NF, Gontijo AF, Pereira MH (2000a) Comparison of feeding behaviour of *Triatoma infestans*, *Triatoma brasiliensis* and *Triatoma pseudomaculata* in different hosts by electronic monitoring of the cibarial pump. J Insect Physiol 46: 1121–1127.

10. Guarneri AA, Pereira MH, Diotaiuti L (2000b) Influence of the blood meal source on the development of *Triatoma infestans*, *Triatoma brasiliensis*, *Triatoma sordida*, and *Triatoma pseudomaculata* (Heteroptera, Reduviidae). J Med Entomol 37: 373–379.

11. Nattero J, Leonhard G, Rodríguez CS, Crocco L (2011) Influence of the quality and quantity of blood ingested on reproductive parameters and life-span in *Triatoma infestans* (Klug). Acta Trop 119: 183–187.

12. Nattero J, Rodríguez CS, Crocco L (2013a) Effects of blood meal source on food resource use and reproduction in *Triatoma patagonica* Del Ponte (Hemiptera, Reduviidae). J Vector Ecol 38: 127–133.

13. Medone P, Balsalobre A, Rabinovich JE, Marti GA, Menu F (2015) Life history traits and demographic parameters of *Triatoma infestans* (Hemiptera: Reduviidae) fed on human blood. J Med Entomol 52: 1282–90.
